# A Case of Thanatophoric Dysplasia Type 2: A Novel Mutation

**DOI:** 10.4274/jcrpe.1703

**Published:** 2015-03-05

**Authors:** Selvi Gülaşı, Aytuğ Atıcı, Yalçın Çelik

**Affiliations:** 1 Mersin University Faculty of Medicine, Department of Pediatrics, Mersin, Turkey

**Keywords:** Thanatophoric dysplasia, FGFR3 gene, Skeletal dysplasia

## Abstract

Thanatophoric dysplasia (TD) is a lethal form of skeletal dysplasia with short-limb dwarfism. Two types distinguished with their radiological characteristics have been defined clinically. The femur is curved in type 1, while it is straight in type 2. TD is known to be due to a mutation in the fibroblast growth factor receptor 3 (FGFR3) gene. We report a male patient who showed clinical findings congruent with TD type 2 and a new mutation in the FGFR3 gene, a finding which has not been reported previously.

## INTRODUCTION

Thanatophoric dysplasia (TD) is a congenital skeletal dysplasia characterized by marked underdevelopment of the skeletal system and short-limb dwarfism. It is the most common form of lethal skeletal dysplasia syndromes. It has first been described by Maroteaux and Lamy in 1967 (1). Its incidence is 1 in 20 000 to 50 000 births and it is an autosomal dominant condition (2). The disease is caused by a mutation in the fibroblast growth factor receptor 3 (FGFR3) gene. This gene is located on the short arm of the fourth chromosome. The mutation leads to reduced apoptosis and increased proliferation due to FGFR3 tyrosine kinase activation (3,4). The most frequently reported mutations are p.R248C, p.S249C and p.Y373C.

Two types of TD have been described. Type 1 TD is characterized by radiological features such as shortness and bowing in the long bones, increased radiolucency and irregularity in long-bone metaphyses, hypoplasia in the pelvic bones and flattening in the acetabular skeleton, hypoplasia in the vertebral corpus and in some cases, a clover-leaf like appearance on the skull (5). The shortness of the bones in type 2 TD is not as significant as in type 1 TD and there is no bowing in the long bones (6). In almost all type 2 TD cases, the skull has a clover-leaf like appearance, related to the early closure of the coronal and lambdoid sutures and the expansion of the temporal lobe (7,8). The clinical signs include macrocephaly, a large anterior fontanel, a prominent forehead, a depressed nasal bridge, protruding prominent eyes, hypertelorism, short extremities, a narrow and bell-shaped thoracic cage, short ribs, a prominent abdomen and hypotonia. Bowing in the femoral bones, typical in type 1 TD, may also be present. A clover-leaf skull is characteristic in type 2 TD (9,10).

Megalencephaly is a common finding in TD. The temporal lobes are expanded throughout all of the axes and the brain appears round. There are fissures that transversely separate the temporal lobe deeply (11). The development of the hippocampus is damaged and there is hypoplasia on the dentate gyrus and Ammon’s horn. Ventriculomegaly is common and has been reported at variable levels. Because ventriculomegaly is commonly limited in the temporal horns, clover-leaf like skull deformity occurs in the cisterna. Due to the foramen magnum being narrow, pressure on the brainstem, hypoplasia on the brainstem and hydrocephaly may possibly develop. Disorders in the cerebellum development have been reported but are not common (12). Patent ductus arteriosus, atrial septal defect and kidney disorders are rarely reported conditions.

Knowing the typical features of TD helps to diagnose it prenatally and allows providing genetic counseling services. TD can be suspected based on clinical and/or prenatal ultrasonographic and radiological findings. However, its distinction from achondroplasia and osteochondrodysplasia, which are other short-extremity dwarfisms, is difficult.

Genetic studies reveal mutations in the FGFR3 gene in both TD types. It is well-known that the FGFR3 signal is the basic organizer of bone growth, chondrocyte differentiation and proliferation. It is recommended to scan exons 7, 10, 15 and 19 in the FGFR3 gene in type 1 TD and to scan exon 15 in type 2 TD. In type 2 TD, Lys650Glu mutation is detected in most of the cases.

The patient we present in this report had clinical symptoms which were congruent with type 2 TD. Genetic examination revealed a T394K mutation on the FGFR3 gene exon 10 area. To our knowledge, this mutation has not been encountered in previously reported cases.

## CASE REPORT

This male infant was born at 38 weeks gestation by c-section to a 36-year-old healthy mother. He was intubated after birth due to signs of respiratory distress. The infant’s neck was short and therefore the intubation was successful only after the third attempt. The orogastric tube could not be placed and there was suspicion of esophageal atresia. Due to the persistence of severe respiratory distress, the newborn patient was referred to our hospital.

Pregnancy history revealed that the mother was not monitored regularly and that due to her hypertensive state, she was given alpha-methyldopa starting from the 6th month of her pregnancy. She underwent only three ultrasonographic examinations and the fetus was reported as normal, but polyhydramnios was detected at each examination. The parents were first cousins and their living six children were healthy.

**Figure 1:**
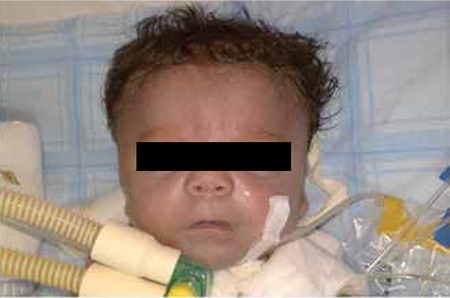
Clover-leaf skull anomaly, flat facial appearance, protrusion of the forehead, protruding prominent eyes, hypertelorism, saddle nose, wide nasal ridge

**Figure 2:**
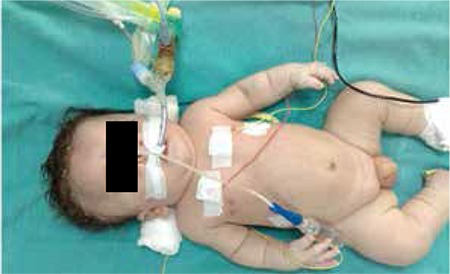
Short limbs particularly in the proximal portions, short fingers, skin folds on limbs, short neck

At admission, the infant’s body weight was 2790 grams (25-50%), his length was 43 cm (50%), his head circumference was 38 cm (>90%). Body temperature was 36.4 ˚C, heart rate was 135/minute, blood pressure was 53/14 mmHg. Oxygen saturation was 85% although he was intubated and on 100% oxygen. Physical examination revealed a large anterior fontanel, prominent forehead, protruding prominent eyes, hypertelorism, depressed nasal root, wide nose ridge, short neck, mild shortness in the extremities, short fingers, skin folds in the extremities, a prominent abdomen and hypotonia (Figures 1 and 2). Breath sounds were decreased bilaterally. There was a II/VIO systolic murmur under the left clavicle. An orogastric tube was inserted and proceeded easily. Esophageal atresia was excluded.

**Figure 3:**
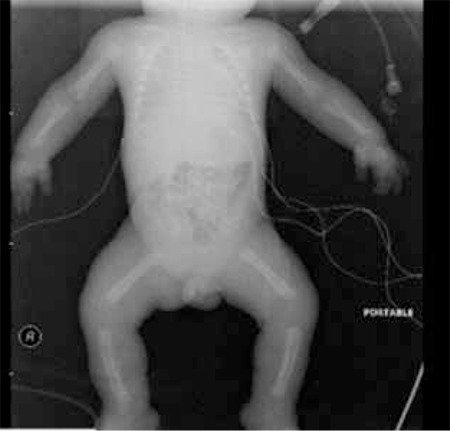
Straight femurs in radiogram of the skeletal system

A tentative diagnosis of TD was considered. A radiogram of the skeletal system showed straight femurs (Figure 3). Transfontanel ultrasound showed that the occipital and temporal horns of the lateral ventricles were large and the sulci were prominent. Echocardiographic examination showed a patent ductus arteriosus and pulmonary hypertension.

**Figure 4:**
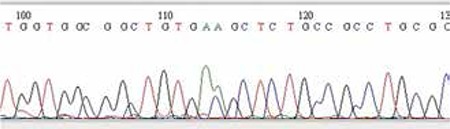
Exon 9, 10, 13 and 15 regions were screened in the DNA samples of the patient; and a heterozygous T394K mutation (from ACG to AAG) was detected in exon 10 of the FGFR3 gene

In the genetic investigation, exons 9, 10, 13 and 15 were scanned. A heterozygous T394K mutation (changing from ACG to AAG) was detected in the exon 10 (Figure 4). The family was informed about the result of the genetic examination and their consent was taken to publish this case including the infant’s pictures.

During the patient’s clinical follow-up, respiratory distress and the need for mechanical ventilation continued and tracheostomy was performed. Upon the request of the family, the patient was transferred to a city hospital on the 75th day of his life.

## DISCUSSION

In this article, we present a case of type 2 TD with a T394k mutation, a finding in the FGFR3 gene which has not been reported previously.

In type 1 TD, the femur is curved like a “telephone receiver”, while, the femur is straight in type 2 TD and the clover-leaf skull deformity is significant. In our case, the femur was observed to be straight in the radiogram and the patient, who also showed the other clinical features of type 2 TD such as short extremities, short fingers, hypotonia, too many skin folds along the short extremities, hypertelorism, a depressed nasal root, a prominent forehead and protruding prominent eyes, was diagnosed to have type 2 TD. The infant was in severe respiratory distress due to pulmonary hypertension, probably related to lung hypoplasia. Inhaled nitric oxide and high frequency oscillatory ventilation treatment was needed. Most infants affected by TD die within the first few hours or days of their lives with respiratory distress. Pulmonary hypoplasia and/or pressure in the brainstem due to small foramen magnum can be the cause of respiratory distress. Some affected infants have been reported to reach childhood age with continuous ventilator treatment (13). Tracheostomy is often necessary. Tracheostomy was applied in our case as well.

In type 2 TD, structural defects may be present in the central nervous system and clinical seizures may occur. Clinical seizures occurred also in our patient and were taken under control with anticonvulsant medication. In the ultrasound examination of our patient, there was ventricular enlargement and prominence of the sulci.

MacDonald et al (13) have reported two cases, a 4-year-9-month-old male and a 3-year-8-month-old female, who underwent tracheostomy and received mechanical ventilation starting from birth. The male infant also had hydrocephalus and required a ventriculoperitoneal shunt and the female infant required a hearing aid due to bilateral hearing loss. Seizures and electroencephalographic disorders have been observed in both cases. Our case underwent tracheostomy and was receiving mechanical ventilation during a follow-up period of 75 days, after which time, he was transferred to another hospital.

In the first trimester of the pregnancy, TD should be suspected if nuchal brightness and extremity shortness are noted in the ultrasonographic examination, In these cases, definitive tests can be done in the second trimester of pregnancy with pathological or molecular methods. In high risk cases, samples for genetic investigation can be obtained by amniocentesis earliest at weeks 15-18 or by chorionic villus samples at weeks 10-12. A tentative diagnosis by these prenatal ultrasound evaluations and genetic tests facilitates an early diagnosis and provision of genetic counseling service.

The mutation in most TD cases is de novo. The parents may be unaffected. In our case, in the genetic investigation performed on the blood sample, exons 9, 10, 13 and 15 in FGFR3 gene were scanned and a heterozygous T394K mutation (changing from ACG to AAG) was detected in the exon 10. To our knowledge, this mutation has not been reported previously (Table 1) (14,15,16,17,18).

## Figures and Tables

**Table 1:**
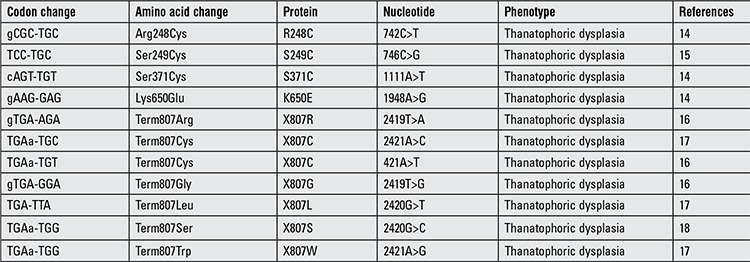
Reported mutations in thanatophoric dysplasia
